# Impact of *TRAF3IP2*, *IL10* and *HCP5* Genetic Polymorphisms in the Response to TNF-i Treatment in Patients with Psoriatic Arthritis

**DOI:** 10.3390/jpm12071094

**Published:** 2022-06-30

**Authors:** Giada De Benedittis, Andrea Latini, Cinzia Ciccacci, Paola Conigliaro, Paola Triggianese, Mauro Fatica, Lucia Novelli, Maria Sole Chimenti, Paola Borgiani

**Affiliations:** 1 Department of Biomedicine and Prevention, Section of Genetics, University of Rome “Tor Vergata”, 00133 Rome, Italy; dbngdi01@uniroma2.it (G.D.B.); latini.andrea@hotmail.com (A.L.); borgiani@med.uniroma2.it (P.B.); 2 UniCamillus–Saint Camillus International University of Health Sciences, 00131 Rome, Italy; lnovelli@hotmail.it; 3 Rheumatology, Allergology and Clinical Immunology, Department of System Medicine, University of Rome “Tor Vergata”, 00133 Rome, Italy; cngpla00@uniroma2.it (P.C.); triggianese@med.uniroma2.it (P.T.); maufat25@gmail.com (M.F.); maria.sole.chimenti@uniroma2.it (M.S.C.)

**Keywords:** psoriatic arthritis, polymorphisms, treatment, TNF-i

## Abstract

Psoriatic arthritis (PsA) is a chronic inflammatory rheumatic disease. The introduction of therapy with biological drugs is promising, even if the efficacy is very variable. Since the response to drugs is a complex trait, identifying genetic factors associated to treatment response could help define new biomarkers for a more effective and personalized therapy. This study aimed to evaluate the potential role of polymorphisms in genes involved in PsA susceptibility as predictors of therapy efficacy. Nine polymorphisms were analyzed in a cohort of 163 PsA patients treated with TNF-i. To evaluate the treatment response, the DAPsA score was estimated for each patient. The possible association between the selected SNPs and mean values of DAPsA differences, at 22 and 54 weeks from the beginning of the treatment, were evaluated by *t*-test. Patients carrying the variant allele of *TRAF3IP2* seemed to respond better to treatment, both at 22 and 54 weeks. This variant allele was also associated with an improvement in joint involvement. In contrast, patients carrying the *IL10* variant allele showed an improvement lower than patients with the wild-type genotype at 54 weeks. Our results suggest that polymorphisms in genes associated with PsA susceptibility could also play a role in TNF-i treatment response.

## 1. Introduction

Psoriatic arthritis (PsA) is a chronic inflammatory rheumatic disease, characterized by articular and periarticular manifestations and frequently associated with psoriasis (PsO) [1]. Its pathogenesis is multifactorial with a strong genetic component [2]. PsA also presents clinical heterogeneity and frequent presence of several comorbidities, such as metabolic syndrome and cardiovascular diseases [3].

The identification of the correct therapy for PsA patients is still a critical issue. The introduction of biological disease-modifying antirheumatic drugs (bDMARDs) seems to be promising in terms of efficacy and safety [4]; the first class of biologic agents approved for PsA is monoclonal antibodies directed against tumor necrosis factor (TNF). TNF is a key proinflammatory cytokine that regulates several inflammatory and immune functions, involved in the pathogenesis of PsA [5]. In particular, TNF induces the expression of the receptor activator of nuclear factor-κB ligand, promoting osteoclastogenesis, and the expression of Dkk-1, inhibiting osteoblastogenesis, both leading to erosion formation [6].

Treatment with TNF-inhibitors (TNF-i) is more promising in improving arthritis, enthesitis, dactylitis and skin, and presents a better efficacy in preventing joint destruction and radiographic progression with respect to therapy with conventional synthetic DMARDs [7]. Despite the encouraging data on these drugs, treatment with TNF-i only induces a significant clinical improvement in approximately 60% of PsA patients [8]. Indeed, some patients completely fail to respond, while others have a loss of efficacy following an initial clinical response. Loss of efficacy is in fact the most major obstacle to the long-term use of TNF-i [9].

It is known that the variability in treatment response could be affected by multiple factors, including the genetic interindividual variability. Indeed, several studies have investigated the effect of single-nucleotide polymorphisms (SNPs) on response to TNF-i [10,11]. These studies have shown associations between genetic variants and treatment response, suggesting a role of genetic variability in the prediction of the efficacy to this treatment [12]. Among the polymorphisms associated to TNF-i treatment response, genetic variants located in genes already known to be involved in PsA susceptibility have also emerged [13,14]. Several studies have reported a role of *HLA* alleles as predictors of response to treatment with bDMARDs in PsA patients [15], while other studies have described significant association of SNPs in the TNF region and TNF-i treatment response [13,16]. Recently, polymorphisms on *TNFAIP3* gene were also observed as associated with TNF-i treatment response in PsA patients [10].

Nevertheless, except for these results, few studies related to pharmacogenetics in PsA patients have been reported in literature [17,18].

In our previous study, we analyzed nine polymorphisms in eight different genes and we showed associations between rs27524 (*ERAP1*), rs1800872 (*IL10*), rs7574865 (*STAT4*), rs6920220 (*TNFAIP3*), rs33980500 (*TRAF3IP2*) polymorphisms and PsA susceptibility [19]. Thus, the current study aimed to investigate the potential role of the same polymorphisms in *ERAP1*, *HCP5*, *IL10*, *MIR146A*, *PSORS1C1*, *STAT4*, *TNFAIP3* and *TRAF3IP2* genes as predictors of efficacy of treatment in a cohort of PsA patients treated with first-line TNF-i, in particular with Etanercept (ETN) and Adalimumab (ADA).

## 2. Materials and Methods

### 2.1. Patients

Blood samples and medical records of PsA patients referred to the Rheumatology Outpatient Clinic of the University of Rome Tor Vergata, Rome, Italy, were retrospectively analyzed (time frame of the enrollment: 2017–2020). Patients among these who begun treatment with ETN or ADA according to the European League Against Rheumatism (EULAR) and/or Group for Research and Assessment of Psoriasis and Psoriatic Arthritis (GRAPPA) guidelines were considered [20,21].

Inclusion criteria were a diagnosis of PsA > 6 months according to the CASPAR classification criteria; being 18 years of age; having active disease, defined as at least three tender joints out of 78 and at least three swollen joints out of 76, despite previous treatment with NSAIDs and/or csDMARDS; inadequate response to at least one conventional synthetic (cs) DMARD, including methotrexate; naïve for bDMARD treatment; and indication to begin ETN or ADA treatment.

Use of concomitant csDMARDs was allowed if dosage had been stable over the previous 3-month period. Although discontinuation or reduction in csDMARD dosage was consented during the study period if the patient’s clinical situation was favorable, increasing the doses was not allowed. Nonsteroidal anti-inflammatory drugs (NSAIDs) were permitted only for a maximum of 3 days a week. Low-dose corticosteroids (daily dose of 10 mg or less of prednisone or equivalent) were permitted. Intra-articular corticosteroid injections were not allowed.

Patients were excluded from the study if they presented prevalent axial disease, showed impairment of hepatic/renal function, alcohol abuse, recent infection (with the last infection > 3 months ago), ongoing history of malignancy (with interval malignancy-free > 5 years) or ongoing pregnancy, and if they had missing or incomplete data in the follow-up visits. Patients received recommended doses of TNF-i: subcutaneous injection of ETN at 50 mg every week or ADA at 40 mg biweekly.

Disease activity and clinical response to therapy were assessed using composite measures, as Disease Activity index for Psoriatic Arthritis (DAPsA) score. DAPsA is a composite score consisting of numbers of tender joints (TJ), numbers of swollen joints (SJ), serum acute-phase response represented by C-reactive protein (CRP), pain visual analogue scale (pVAS) and patient global assessment (gVAS) [22].

The clinical and laboratory findings were evaluated at baseline and every 3 months from the start of TNF-i therapy. Data concerning DAPsA low-disease activity and remission were registered at 6 months and 1 year after the beginning of the TNF-i treatment. Laboratory assessment included CRP assessed by nephelometry (normal range, 0 ± 3 mg/L). Peripheral blood samples were obtained at the time of the first medical evaluation in order to perform the genetic analyses. Samples were stored at −80 °C until they were analyzed.

The drug’s safety was evaluated by assessing adverse events and via standard laboratory testing.

Written informed consent was obtained from patients. The study protocol was approved by the local ethics committee (Approval No. RS186/16, 9 November 2016) of the Policlinico Tor Vergata in Rome (Italy).

### 2.2. DNA Extraction and Genotyping

Genomic DNA extraction from peripheral blood mononuclear cells by Qiagen blood DNA mini kit and genotyping analysis by allelic discrimination assay with TaqMan technology were described in a previous paper [19]. We investigated the following SNPs: rs27524 (*ERAP1*), rs3099844 (*HCP5*), rs1800872 (*IL10*), rs2910164 (*MIR146A*), rs2233945 (*PSORS1C1*), rs7574865 (*STAT4*), rs6920220 and rs2230926 (*TNFAIP3*) and rs33980500 (*TRAF3IP2*).

### 2.3. Statistical Analysis

We evaluated a possible association between the selected SNPs and the response to therapy at 22 and 54 weeks from the beginning of the TNF-i treatment, using as a clinical parameter the values of DAPsA. Differences in genotype frequencies between groups of patients that achieved and did not achieve DAPsA low-disease activity and DAPsA remission were performed by Pearson’s χ^2^ test and the possible contribute of each single polymorphism on TNF-i treatment response was evaluated by univariate analysis. The mean differences of DAPsA values between the genotypic classes for each analyzed SNP were compared by a *t*-test. A multivariate logistic regression analysis was used to evaluate the contribution of each investigated genetic variant in the TNF-i treatment response, including as independent variables all analyzed SNPs, sex and age. Two-tailed *p* values less than 0.05 were considered statistically significant. All statistical analyses were performed by the SPSS program ver. 19 (IBM Corp, Armonk, NY, USA).

## 3. Results

### 3.1. Clinical Characteristics of Patients

The study included 160 PsA patients of Caucasian origin, of whom 43.8% (*n* = 70) were treated with ETN and 56.3% (*n* = 90) were treated with ADA. Patients’ clinical and demographic data are summarized in Table 1.

Patients had longstanding disease in 72.5% of the cases. CRP was positive in 8.12% of patients. Mean DAPsA at the beginning of the treatment was 24.95 ± 14.24. Patients with PsA receiving concomitant csDMARDs comprised 64.37%.

After 6 months of TNF-i treatment, DAPSA low-disease activity was achieved in 69.6% and DAPsA remission in 16.5% of the whole PsA population. After 1 year of treatment, DAPSA low-disease activity was achieved in 74.3% and DAPsA remission in 32.2% of the whole PsA population.

We observed two dropout patients (1.3% of the whole study population) at T22 and 9 at T54 (5.6%) because of adverse events (*n* = 2), secondary failure (*n* = 6) and concomitant conditions (*n* = 1). No differences in demographic, clinical data and response to treatment were detected between subgroups of patients treated with ETN or ADA.

### 3.2. Associations of Genetic Variants with Response to TNF-i Treatment

We investigated the possible role of nine SNPs in eight candidate genes on TNF-i treatment response. Firstly, for each single polymorphism, we compared the genotypes distribution in relation to DAPsA low-disease activity and DAPsA remission (achieved vs. not achieved) at 22 and 54 weeks after the treatment starting. We observed no significant association between the analyzed SNPs and the achievement of DAPsA low-disease activity and DAPsA remission at T22 and T54 (data not shown), neither in the whole cohort nor in each TNF-i drug subgroup.

In a second step, for each PsA patient, the response to the treatment was evaluated considering the changes of DAPsA values at the follow up at 22 (ΔT22) and at 54 weeks (ΔT54) with respect to the beginning of treatment. Therefore, we compared the mean values of DAPsA differences (ΔT22 and ΔT54) in the different genotypic classes for each SNP analyzed. We tested the additive and recessive models without observing significant associations (data not shown), while using the dominant model, we observed several associations (Table 2).

We observed that *TRAF3IP2* SNP was associated with TNF-i treatment response in PsA patients. In particular, the mean DAPsA values of patients carrying the variant allele show a statistically greater decrease than the wild-type patients, both at 22 (*p* = 0.032) and 54 weeks (*p* = 0.019).

*IL10* SNP, instead, was associated with TNF-i response only at 54 weeks of treatment (*p* = 0.031). PsA patients carrying the *IL10* variant allele decreased their mean DAPsA value further than patients with the wild-type genotype, suggesting that patients with the variant allele have a lower improvement after treatment.

Lastly, *HCP5* polymorphism showed a mean difference of DAPsA values between genotypes for both follow-up periods, even if these differences did not reach a statistical significance (*p* = 0.068 and *p* = 0.086).

**Figure 1:**
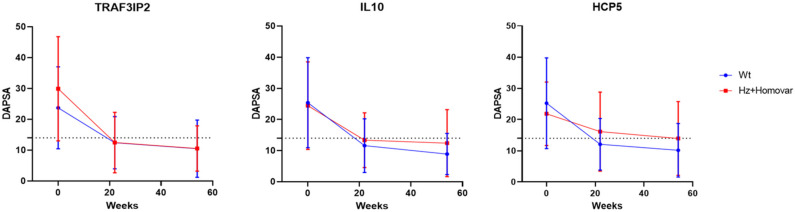
Mean differences of DAPsA values in the genotypic classes for *TRAF3IP2*, *IL10* and *HCP5* SNPs. DAPsA = Disease Activity Index for PsA; Wt = wild-type genotype; Hz = heterozygous genotype; Homo var = homozygous genotype. The dotted line indicates 15 DAPsA value corresponding to low disease activity.

The mean differences of DAPsA values in the genotypic classes for these three SNPs are represented in Figure 1. For the other SNPs, no significant differences between the genotypic classes were observed.

**Figure 2:**
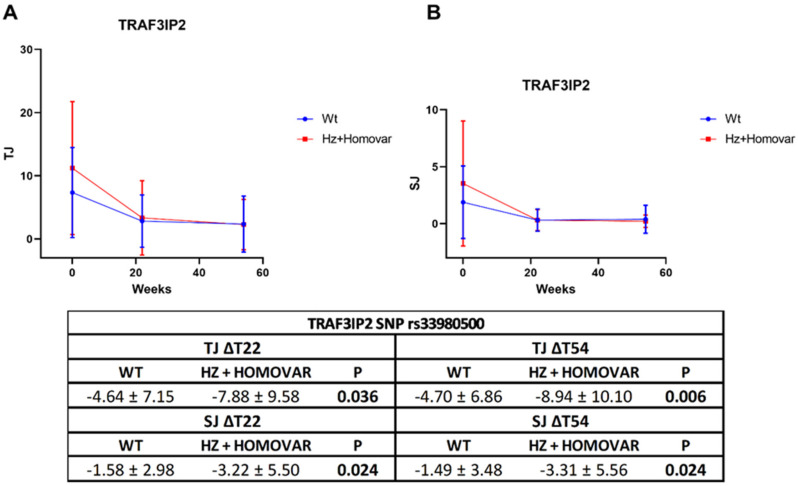
Mean differences of TJ (**A**) and SJ (**B**) values in the genotypic classes for *TRAF3IP2* SNP. TJ = tender joint; SJ = swollen joint; Wt = wild-type genotype; Hz = heterozygous genotype; Homo var = homozygous genotype.

In light of the associations observed for *TRAF3IP2* and *IL10* SNPs, we performed the same analysis considering the mean differences for each single component of the DAPsA score. Interestingly, the variant allele of *TRAF3IP2* SNP resulted as associated with a better response of joint involvement. Indeed, the number of TJ and SJ decreased more in patients carrying the variant allele, both at 22 and 54 weeks (Figure 2).

We also performed a multivariate regression analysis to better evaluate the contribution of each investigated genetic variant in the TNF-i treatment response (Table 3).

This analysis was performed with a stepwise method and it confirmed the involvement of *TRAF3IP2* (*p* = 0.016) and HCP5 (*p* = 0.035) polymorphisms in the TNF-i response after 22 weeks and of *TRAF3IP2* (*p* = 0.007), IL10 (*p* = 0.022) and *HCP5* (*p* = 0.036) polymorphisms after 54 weeks. The two final models explain about 6% and 11% of the variability in TNF-i treatment response at 22 and 54 weeks, respectively.

Subsequently, we repeated the analysis evaluating each single TNF-i (Table 4).

We observed that the associations with *TRAF3IP2* SNP were drug-specific. Indeed, the patients carrying the *TRAF3IP2* variant allele seem to respond better to the treatment, both at 22 (*p* = 0.043) and 54 weeks (*p* = 0.029), only when treated with ETN. With regard to IL10 SNP, instead, we confirmed that patients carrying the variant allele showed a worse response at 54 weeks only for ADA treatment (*p* = 0.016). Lastly, the *STAT4* SNP variant carriers showed a minor decrease in mean DAPsA values compared to patients with wild-type genotype at 22 weeks of ADA treatment. Indeed, the variant alleles resulted as associated with a worse response (*p* = 0.006).

**Figure 3:**
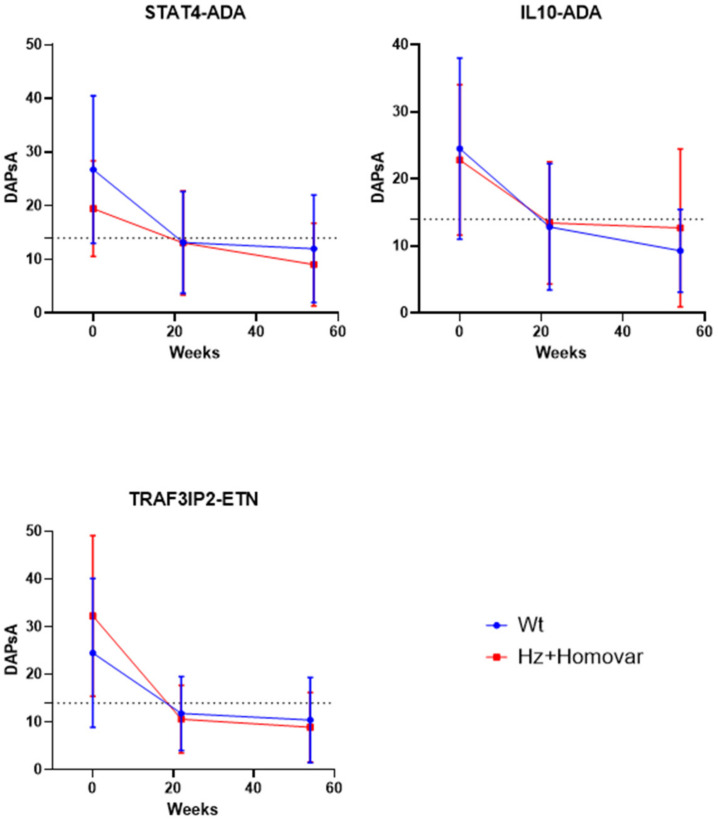
Mean differences of DAPsA values for single TNF-inhibitor treatment in the genotypic classes for *STAT4*, *IL10* and *TRAF3IP2* SNPs. ADA = adalimumab; ETN = etanercept; DAPsA = Disease Activity Index for PsA; Wt = wild-type genotype; Hz = heterozygous genotype; Homo var = homozygous genotype. The dotted line indicates 15 DAPsA value corresponding to low disease activity.

The mean differences of DAPsA values in the different genotypic classes for these three SNPs are represented in the Figure 3.

## 4. Discussion

In this study, we evaluated the potential role of polymorphisms in eight different genes, already investigated in relation to PsA susceptibility in our previous study [19], in the response to ETN and ADA treatment in a cohort of PsA patients.

The results of the present study showed the association of the investigated SNPs rs33980500 (*TRAF3IP2*), rs1800872 (*IL10*) and rs3099844 (*HCP5*) with TNF-i response.

We observed that patients carrying the variant allele of *TRAF3IP2* SNP seem to respond better to the treatment, but when we stratified the patients by the drug, this result was found only in the subgroup of patients treated with ETN. Moreover, the variant allele of rs33980500 SNP on *TRAF3IP2* resulted as associated with a better response of joint involvement. In a previous study, we had also described an association of the variant allele in *TRAF3IP2* gene with a higher number of tender/swollen joints and a higher DAPsA score [19]. The patients carrying this variant allele had a higher DAPsA value at the beginning of therapy compared to patients carrying the wild-type allele, but at 22 and 54 weeks of treatment we did not observe a difference in DAPsA value between the two genotype groups. Therefore, the greater decrease in mean DAPsA values observed in patients carrying the variant allele could be attributed to the high disease activity at the beginning of therapy. *TRAF3IP2* encodes for Act1, which interacts with TRAF proteins, and it is reported that the rs33980500 SNP decreases the binding with TRAF2, TRAF3 and TRAF6 [23]. Previously, we have highlighted the involvement of rs33980500 SNP in the response to TNF-i treatment in rheumatoid arthritis (RA) patients [24]. Recently, *TRAF3IP2* has been investigated in relation to TNF-i response, as well as in psoriasis and PsA patients, by Ovejero-Benito et al., who reported no significant association between polymorphisms located on this gene and response to anti-TNF drugs [10].

Regarding *IL10*, patients with the variant allele seem to have a lower improvement after treatment compared to patients carrying the wild-type allele at 54 weeks of therapy. Indeed, we observed that the variant allele was associated with a minor decrease in mean DAPsA value. This result was replicated only in the subgroup of patients treated with ADA. The patients carrying the variant allele of *IL10* had a higher DAPsA value at 54 weeks of treatment compared to patients carrying the wild-type allele, despite DAPsA values being similar in the two genotype groups at the beginning of therapy. Therefore, we suggest that these variants could contribute to the TNF-i efficacy.

IL10 gene encodes for a pleiotropic anti-inflammatory cytokine [25] and several polymorphisms on this gene have been identified with possible consequences on the response to TNF-i treatment in other autoimmune diseases, such as RA [26,27] and inflammatory bowel disease [28]. Our investigated SNP rs1800872 is localized in the promoter region and the variant allele is associated with increased production of the cytokine [29]. Schotte et al. described an association of genotypes that regulate IL-10 production, including rs1800872, with ETN response in RA patients [30]. According to our data, they suggest that a constitutionally high IL-10 production may negatively affect the response to anti-TNF therapy.

In addition, for *HCP5* polymorphism, we showed a lower decrease in DAPsA value in patients carrying the variant allele for both follow-up periods, but only in multivariate regression analysis. In fact, these patients had a higher DAPsA value at 54 weeks of treatment compared to patients carrying the wild-type allele, despite us not observing a difference in DAPsA value between the two genotype groups at the beginning of therapy. The *HCP5* gene is located in the major histocompatibility complex class I region and codes for a long noncoding RNA involved in many autoimmune diseases [31,32]. In particular, the polymorphism rs2395029 on this gene was described as associated with psoriasis and PsA [27,33]. Despite this association with susceptibility to PsA, there are no studies regarding the possible involvement of this gene in the response to anti-TNF therapy.

We found that the variant allele of *STAT4* (rs7574865) was associated with a worse response at 22 weeks of ADA treatment compared to patients carrying the wild-type allele. *STAT4* SNPs have been already described as associated with susceptibility to other autoimmune conditions, such as RA [34], and several studies have reported a correlation between the variant allele of rs7574865 and higher levels of *STAT4* mRNA [35]. It is known that STAT4, expressed by T cells, induces the production of interferon and IL-17, and its increase could influence the response to ADA therapy [36]. Moreover, in patients carrying this variant allele of STAT4, the availability of efficacious drugs that directly target JAK/STAT-dependent inflammation, as Jak-I, could disrupt several downstream signal-transduction axes, with subsequent positive effects at the systemic and joint levels [37]. Knowledge of these signal-transduction axes is, therefore, important for understanding how each cytokine/node is dependent on or independent from others.

The main limitations of our study were (1) the exclusion of patients with axial involvement due to the lack of a definition of axial-PsA as main manifestation of the disease; (2) the small number of patients affected by dactylitis or enthesitis, which prevented us from assessing as far as that manifestation was concerned; (3) the relatively limited number of patients included, and the subdivision among patients treated with ADA or ETN, which may have influenced the statistical significance of the results.

To conclude, our findings suggest that SNPs in genes associated with disease susceptibility might be involved also in response to TNF-i treatment. If our findings will be confirmed in further studies and larger cohorts, these polymorphisms could be useful to define a genetic profile associated with the response to this treatment. The road to personalized medicine in PsA is promising and surely the role of genetic biomarkers to support the treatment choice is challenging and encouraging.

## Figures and Tables

**Table 1:** Clinical data of the 160 PsA patients.

Sex (% of males)	50.9
Age (mean ± SD)	59.9 ± 12.48
Age at diagnosis (mean ± SD)	44.7 ± 12.47
TJ (mean ± SD)	8.04 ± 7.91
SJ (mean ± SD)	2.23 ± 3.84
CRP (mean ± SD)	1.97 ± 5.23
pVAS (mean ± SD)	7.12 ± 6.44
gVAS (mean ± SD)	6.79 ± 5.19
DAPsA (mean ± SD)	24.95 ± 14.24
Enthesitis (%)	17.3
Dactylitis (%)	11.7
Erosions (%)	11.1
Psoriasis (%)	51.3

Quantitative data are expressed as mean and standard deviation (SD); dichotomous data are expressed as a percentage. TJ = numbers of tender joints; SJ = numbers of swollen joints; CRP = C-reactive protein; pVAS = patient pain assessment; gVAS = patient global assessment; DAPsA = Disease Activity Index for PsA.

**Table 2:** Association analysis, according to the dominant model, for *STAT4*, *TRAF3IP2*, *TNFAIP3*, *MIR146A*, *PSORS1C1*, *IL10*, *HCP5* and *ERAP1* polymorphisms and response to TNF-inhibitors treatment in PsA patients.

	ΔT22			ΔT54		
***STAT4* SNP**	**WT**	**HZ + HOMOVAR**	***p***	**WT**	**HZ + HOMOVAR**	***p***
rs7574865	−13.50 ± 14.34	−11.52 ± 14.27	0.39	−14.46 ± 14.53	−14.54 ± 14.82	0.97
***TRAF3IP2* SNP**	**WT**	**HZ + HOMOVAR**	***p***	**WT**	**HZ + HOMOVAR**	***p***
rs33980500	−11.36 ± 13.08	−17.42 ± 17.53	**0.032**	−13.11 ± 13.35	−19.97 ± 17.73	**0.019**
***TNFAIP3* SNP**	**WT**	**HZ + HOMOVAR**	***p***	**WT**	**HZ + HOMOVAR**	***p***
rs6920220	−12.93 ± 13.69	−12.12 ± 15.29	0.73	−14.81 ± 14.84	−14.26 ± 14.33	0.82
rs2230926	−12.76 ± 14.51	−10.21 ± 9.83	0.58	−14.77 ± 14.80	−10.81 ± 10.10	0.43
***MIR146A* SNP**	**WT**	**HZ + HOMOVAR**	***p***	**WT**	**HZ + HOMOVAR**	***p***
rs2910164	−12.28 ± 15.09	−13.01 ± 13.18	0.75	−14.90 ± 14.06	−14.06 ± 15.28	0.73
***PSORS1C1* SNP**	**WT**	**HZ + HOMOVAR**	***p***	**WT**	**HZ + HOMOVAR**	***p***
rs2233945	−12.43 ± 13.33	−12.94 ± 16.24	0.84	−14.12 ± 13.24	−15.27 ± 17.19	0.65
***IL10* SNP**	**WT**	**HZ + HOMOVAR**	***p***	**WT**	**HZ + HOMOVAR**	***p***
rs1800872	−13.89 ± 14.74	−11.09 ± 13.59	0.22	−16.92 ± 14.04	−11.79 ± 14.78	**0.031**
***HCP5* SNP**	**WT**	**HZ + HOMOVAR**	***p***	**WT**	**HZ + HOMOVAR**	***p***
rs3099844	−13.22 ± 14.61	−5.69 ± 6.47	0.068	−15.15 ± 14.77	−7.90 ± 10.43	0.086
***ERAP1* SNP**	**WT**	**HZ + HOMOVAR**	***p***	**WT**	**HZ + HOMOVAR**	***p***
rs27524	−13.25 ± 15.54	−12.26 ± 13.91	0.71	−14.78 ± 13.42	−14.38 ± 15.15	0.88

“WT” indicates the homozygous genotype for the wild-type allele; “HZ” indicates the heterozygous genotype; “HOMOVAR” indicates the homozygous genotype for the variant allele. “ΔT22” and “ΔT54” indicates the mean values of DAPsA differences at 22 and 54 weeks from the beginning of the TNF-i treatment. *p* = *p* value evaluated by *t*-test. Significant *p* values are reported in bold.

**Table 3:** (**a**) Final model of multiple linear regression analysis by stepwise method for ΔT22 as dependent variable. (**b**) Final model of multiple linear regression analysis by stepwise method for ΔT54 as dependent variable.

(**a**)				
**Independent Variables**	**Beta Standardized Coefficient**	**t-Statistics**	***p***	**R^2^**
***TRAF3IP2*** rs33980500	−0.196	−2.445	**0.016**	0.061
***HCP5*** rs3099844	0.171	2.134	**0.035**	
(**b**)				
**Independent Variables**	**Beta Standardized Coefficient**	**t-Statistics**	***p***	**R^2^**
***TRAF3IP2*** rs33980500	−0.220	−2.727	**0.007**	0.110
***HCP5*** rs3099844	0.186	2.320	**0.022**	
***IL10*** rs1800872	0.171	2.123	**0.036**	

Variables included in the analysis: age, sex, rs7574865 (*STAT4*), rs33980500 (*TRAF3IP2*), rs6920220 and rs2230926 (*TNFAIP3*), rs2910164 (*MIR146A*), rs2233945 (*PSORS1C1*), rs1800872 (*IL10*), rs3099844 (*HCP5*) and rs27524 (*ERAP1*).

**Table 4:** Association analysis (according to dominant model) between *TRAF3IP2*, *STAT4* and *IL10* and response to single TNF-i treatment in PSA.

**ETANERCEPT**	**DAPsA ΔT22**			**DAPsA ΔT54**		
***TRAF3IP2* SNP**	**WT**	**HZ + HOMOVAR**	***p***	**WT**	**HZ + HOMOVAR**	***p***
rs33980500	−12.59 ± 14.84	−21.67 ± 19.22	**0.043**	−12.84 ± 16.34	−23.84 ± 18.54	**0.029**
**ADALIMUMAB**	**DAPsA ΔT22**			**DAPsA ΔT54**		
***STAT4* SNP**	**WT**	**HZ + HOMOVAR**	***p***	**WT**	**HZ + HOMOVAR**	***p***
rs7574865	−13.58 ± 12.17	−6.45 ± 10.61	**0.006**	−14.73 ± 12.94	−11.81 ± 9.99	0.274
***IL10* SNP**	**WT**	**HZ + HOMOVAR**	***p***	**WT**	**HZ + HOMOVAR**	***p***
rs1800872	−11.95 ± 13.22	−9.37 ± 10.51	0.319	−16.50 ± 12.76	−10.33 ± 10.04	**0.016**

“WT” indicates the homozygous genotype for the wild-type allele; “HZ” indicates the heterozygous genotype; “HOMOVAR” indicates the homozygous genotype for the variant allele. “ΔT22” and “ΔT54” indicates the mean values of DAPsA differences at 22 and 54 weeks from the beginning of the TNF-i treatment. *p* = *p* value evaluated by *t*-test. Significant *p* values are reported in bold.

## Data Availability

Not applicable.
